# Dialysis headache

**DOI:** 10.1590/0004-282X-ANP-2022-E002

**Published:** 2022-03-12

**Authors:** Mario Fernando Prieto PERES

**Affiliations:** 1 Universidade de São Paulo, Faculdade de Medicina, Hospital das Clínicas, Instituto de Psiquiatria, São Paulo SP, Brazil. Universidade de São Paulo Faculdade de Medicina Hospital das Clínicas São Paulo SP Brazil

Headache in dialysis is the rule, not the exception^1^. Although a common and debilitating experience in patients undergoing the procedure, little is known about its mechanisms. In this edition, Melo et al.^2^ is shedding some light in the understanding of this condition by studying 100 consecutive dialytic patients with structured questionnaires and transcranial doppler ultrasonography.

Although the International Classification of Headache Disorders (ICHD-3)^3^ makes “Dialysis Headache” seem too simple, broad, and obvious, without any attempt of distinguishing different patterns within this context, the authors found migraine or tension-type headache in three quarters of the studied population, but headaches starting or worsening during dyalisis and/or resolving within 72 hours occurring in only half.

Two new aspects have been shown in this paper. Quality of life was significantly worse in headache patients; therefore, more attention should be given to the topic. The neurovascular mechanism was studied with transcranial doppler of the middle cerebral artery bilaterally, comparing blood flow and vascular resistance. Dialysis headache is neurovascular, due to cerebral vasodilation. It has been previously proposed that nitric oxide might be involved^4^, an assumption confirmed by Melo et al.

Implications arise from these findings, in therapy and classification. Should we pretreat patients so that dialysis can be prevented? If so, what kinds of therapies could be started? Since CGRP is involved, would Anti-CGRP Monoclonal Antibodies help? Are patients responsive to triptans? Hopefully the future will tell and guide treatments by further clinical trials in this setting.

In terms of classification, if the tension-type or migraine pattern prevails, aren’t we talking about a primary headache trigger? It seems more logical thinking “dialysis headache” as a trigger rather than a secondary headache, as it is currently defined in the ICHD-3. More research is welcome in the area.
